# Teaching and learning with instructional humor: a review of five-decades research and further direction

**DOI:** 10.3389/fpsyg.2025.1445362

**Published:** 2025-01-29

**Authors:** Weichen Zhou, Jun Choi Lee

**Affiliations:** ^1^Faculty of Cognitive Sciences and Human Development, University of Malaysia Sarawak, Kota Samarahan, Sarawak, Malaysia; ^2^School of Teacher Education, Shaoxing University, Shaoxing, Zhejiang, China

**Keywords:** humor, education, instructional humor, learning performance, comprehensive review

## Abstract

The discussion about how to use instructional humor in class to promote teaching and learning efficiency has always been a concern of researchers in recent decades. The present project summarizes extant studies on instructional humor and provides a detailed review of research findings. First, the definition and classification of instructional humor are overviewed. Then, the study introduces three theoretical frameworks, namely Instructional Humor Processing Theory (IHPT) and other two alternative models, which, respectively, based on Self-Determination Theory (SDT) or from an integrative perspective of cognition and affection, explaining how humor works in education settings. Based on the theoretical clarification of instructional humor, the paper further reviews existing empirical evidence regarding teachers’ use of humor in class and its impact on students’ learning, with emphasis on explaining inconsistencies in previous conclusions and identifying limitations in extant relevant works. The detailed analysis and comparison of previous results regarding instructional humor offer potential directions for further relevant research. Finally, the study concludes with feasible advice for teachers to maximize the positive benefits of humor in class.

## 1 Introduction

Euclid, a famous ancient Greek mathematician who is known as the father of geometry, once was teaching the first theorem of geometry in class. He found a student lost focus and kept on whispering to desk mate and doing petty actions. Euclid paused his lecture and reminded the distracted student to swift back his attention to class. Unexpectedly, the student suddenly stood up and asked: “What on earth was the concrete benefits of learning geometry.” Euclid did not answer the question after a moment of silence, instead, he ordered his servant and said: “Please get some money and give it to this gentleman, he would never study without ‘concrete benefit’.” The student was overwhelmed with shame. Euclid resolved the surprised situation in class and corrected the wrong thoughts of the student by a humorous way instead of criticizing him directly. The story indeed proposes an essential question about whether adopting humor in educational settings enhances the process and results of teaching and learning. This ancient but novel issue has been concerned by researchers and educators in recent decades.

The practical usage of humor in education, known as instructional humor (or teaching humor), is a subject of study in educational research. It is often referred to as teachers’ humor in literature, given that the initiation of humorous interactions predominantly originates from educators rather than students. Consistent with the story of Euclid quoted above, several researchers suggest that teachers should integrate humor into their lessons. Numerous studies have highlighted the significant advantages of instructional humor, particularly its positive impact on learning outcomes (Kaplan and Pascoe, 1977; Lujan and Dicarlo, 2016; Ziv, 1988) and the creation of a conducive learning environment characterized by positive classroom vibrations (Mayo, 2010; Petraki et al., 2016; Stuart and Rosenfeld, 1994). These cognitive and social advantages of instructional humor, commonly advocated by studies pro, offer valuable insights for enhancing teaching practices through the strategic application of humor (Bell and Pomerantz, 2016). However, some other researchers argue that instructional humor may not enhance learning performance and may, in some instances, impede the process of knowledge acquisition (Bolkan et al., 2018; Conkell et al., 1999; Weaver et al., 1988). Additionally, the incorporation of humor into classroom settings has been linked to the emergence of negative emotional responses among students (Bieg et al., 2017; Gao et al., 2022; Gorham and Christophel, 1990). These findings suggest that the effectiveness of instructional humor as a pedagogical strategy is not unequivocal (Teslow, 1995). The divergent outcomes observed across studies can be attributed to variations in research methodologies and the specific types of humor examined. This discrepancy highlights the challenges faced by educators in fully using humor to enhance teaching and learning outcomes, underscoring the necessity for comprehensive review of existing relative literatures to provide directions for further nuanced research in this area.

In short, the question of how instructional humor is conducted in education settings and whether it is effective remains unclear. To address the above-mentioned issues, the present study purposes to conduct a comprehensive review of literature from the past five decades to advance our knowledge of instructional humor. According to the summary of existing articles, this paper further aims to discuss the contradictory findings in prior research and identify areas for future research within this field. To achieve these objectives, the current study performed a structured review of existing literature, beginning with a detailed exploration of the concept of instructional humor, including its definitions, categorizations, and theoretical framework. Subsequently, we discuss empirical studies’ findings on the application of humor by educators and its impacts on students’ learning. Lastly, we provide a comprehensive summary evaluating the overall efficacy of instructional humor and recommendations for future research directions to refine the strategies for incorporating humor into educational practices effectively.

## 2 Search method

The concept of instructional humor transcends the confines of conventional disciplinary boundaries and presents challenges to review work. Findings from scholarship on humor in the field of psychology are also useful in educational background for the traceability of instructional humor, such as the theory of humor interprets the reason we laugh in a humorous class (e.g., Suls, 1972) and the framework of humor classification guides its usage by educators in class (e.g., Martin et al., 2003). Additionally, education involves a wide range of subject and does not only occur in traditional classroom. For example, humor integrated in video lectures in online learning environment (e.g., Wang and Chen, 2022), clinical practical instruction (e.g., Chiarello, 2010), and the daily guidance and conversation of doctoral supervision (Kobayashi and Berge, 2022) is also kinds of education outside the formal class-instruction. In consequence, we should use a broad lens not limited to the field of education and traditional classroom while reviewing literature on the topic of instructional humor. The research achievement examined strictly in the field of education must be connected to the broader academic scholarship on humor.

The present work adopted an iterative process of search and evaluation to follow instructional humor and generate a coherent review. The review began by searching for the term “instructional humor” (and alternate spellings, e.g., teaching humor, teacher humor, instructor humor) in abstract of peer-reviewed journal articles in the online literature database such as Web of Science, Elsevier, and Springer Link, without limiting publication year. Another term “humor” was also used but the topic was limited related the field of education (e.g., humor & class, humor & learning, humor & student etc.). We included the articles for further evaluation according to the following criteria with no limitations on country and nationality: studies conducted in schools or other education settings (online and offline), studies related to students’ perception and preference of humor used by teachers, and studies on the topic of teachers’ difference in humor usage. These topics narrowly and broadly had clear implications for educational contexts and processes which contains instructional humor. Empirical and nonempirical studies were included, as were accessible published book and chapters. Besides, references in these works (especially review literature) were searched and obtained for additional relevant citations from a variety of disciplinary publications, and then evaluated.

Based on these criteria, the following areas of research on instructional humor were excluded from this review. Studies those belong to single-subject on psychology, for example, an investigation examined the relationship between humor styles, gelotophobia and self-esteem among university students (Hiranandani and Yue, 2014). These studies are marginally relevant to the use of humor in education although they are conducted in class and students. Some studies on the field of humor comprehension and appreciation were excluded as same because they reveal the individual physiological basis of humor processing from cognitive neural perspective instead of the view of education (e.g., Bartolo et al., 2006). In addition, research that are wholly focus on students’ perception of humorous materials were also beyond consideration, for instance, how students examined humor used in characterization in literatures (Onofrey, 2006). Because they deal solely with the attitudes of students rather than how students prefer humor in texts or other kind of learning materials in order to guide the teachers design them in school curricula. However, studies that involves how students perceive their teachers and learning as the result of humor usage were reviewed because of their implication on pedagogy.

In total, about 140 related works were reviewed in this article and contributed to two major topics of instructional humor, regarding its theoretical overview and practical usage in education. The purpose of these search methods and criteria was not to access every scholarly and relevant work on instructional humor. Because instructional humor exists in a wide range of academic disciplines and outside of traditional instructional mode, resulting that some relevant literatures were not found by the mentioned ways. Nevertheless, the reviewed works were sufficient to provide a detailed introduction of instructional humor, and framework into which these uncaptured literatures could most likely be categorized. These achievements further illustrate trends and future scholarly avenues for instructional humor in education field.

## 3 The overview of instructional humor

This section provides a concise overview of various aspects related to instructional humor, serving as a basis for the subsequent review. It begins with the definition and classification of instructional humor, addressing the fundamental question of identifying humorous attempts in an educational context. Then, we present an overview of theories related to instructional humor, discussing its function in educational settings. Overall, these theoretical perspectives facilitate the understanding and application of this distinct strategy in teaching.

### 3.1 Definition of instructional humor

The concept of “instructional humor” merges two distinct elements: “instruction” and “humor,” necessitating a definition that encompasses both dimensions. Thus, it is important to explore the essence of humor, given that instructional humor inherently refers to the broader category of humor (Banas et al., 2011). The term “humor” originates from the humoral theory in ancient Greek medical science, with its roots in Latin referring to body fluids responsible for lubrication (Garcia-Orozco et al., 2023). Ancient Greek physician Hippocrates posited that human temperament was governed by the balance of four specific humors within the body (Hoppe, 2019). Consequently, “humor” has evolved to denote temperament and disposition beyond its original connotation. The transformation of “humor” to signify something amusing or witty can be traced back to the late 16th century, notably with Ben Johnson’s comedy, *Every Man in His Humour*. This play featured characters with humorous temperaments and traits, contributing to the term’s association with wit and amusement (Craig, 2001). Today, “humor” is commonly understood as an attribute that elicits laughter through entertaining and amusing expressions in behavior, speech, and writing (Hornby, 2004). It involves the playful interaction of multiple, often incongruous meanings in expressions, intentionally or unintentionally, leading to laughter (Martin, 2007). This interaction, characterized by non-serious and amusing content, can manifest through both verbal and nonverbal communication (Booth-Butterfield and Booth-Butterfield, 1991).

Instructional humor, within the context of educational settings, is distinct due to its integration with the objectives and constraints associated with the term “instructional.” “Instruction” involves the deliberate act of teaching, aimed at fulfilling curriculum goals and facilitating the learner’s mastery of knowledge and skills (Flake, 2017). This term, rooted in Late Middle English as “instruccioun,” conveys the act of providing structure and guidance (Random, 1998). Thus, the adjective “instructional” specifies that such humor is utilized deliberately in classroom settings to support and enhance the educational process. As outlined by Bell and Pomerantz (2016), instructional humor is employed with the intention of achieving specific learning objectives through clever, well-founded, yet light-hearted and engaging methods. These methods span a wide array of instructional interactions, including student engagement, the use of educational materials, and the interpretation of course content, all designed to provoke laughter and thereby enrich the learning experience (Petraki et al., 2016). Thus, instructional humor can be characterized as an interactive tactic that leverages both verbal and non-verbal expressions to render educational encounters more engaging, with the ultimate goal of enhancing the efficacy of teaching and learning.

### 3.2 Classification of instructional humor

The classification of instructional humor is complex and varies according to different taxonomies. Some researchers prefer to classify humor based on its forms, a straightforward approach to categorization. Berger (1976) identified 45 specific humor techniques, organizing them into four broad categories: language, logic, identity and action, which correspond to verbal, ideational, existential and physical expressions of humor, respectively. Building on this framework, Bryant et al. (1979) conducted a study on college teachers, categorizing instructional humor into six types: jokes, riddles, puns, funny stories, humorous comments, and others (e.g., mimicking sounds like Donald Duck). This study also considered whether the humor was prepared or spontaneous and evaluated its impact on instructional objectives and potential to disparage others, though it did not differentiate humor types based on these distinctions. Qian (2004) proposed a division between language humor and contextual humor, focusing on linguistic aspects. Language humor involves the humorous characteristics of the language itself, such as wit and allegory, while contextual humor depends on the interplay between words and their contexts. However, these studies predominantly focus on verbal humor, highlighting a gap in research concerning non-verbal humor. To address this, Bolkan et al. (2018) explored text-based instructional humor, identifying two types: exaggeration and absurdity. Exaggeration relates to linguistic rhetoric, and absurdity is linked to logical incongruities. Moreover, Gorham and Christophel (1990) argued for a classification that includes both the forms and targets of humor. They identified 13 specific types of humor used by teachers, focusing on the humor’s targets, such as the teachers themselves, students, or the lessons. Neuliep (1991) expanded this classification to 20 items, organizing them into five categories: teacher-targeted humor, student-targeted humor, untargeted humor, external source humor, and nonverbal humor, based on an examination of high school classrooms. This study also described instructional humor in relation to course content and included physical humor, such as making funny faces, providing a comprehensive overview of how humor can be integrated into educational settings.

The classifications previously outlined focus on the inherent characteristics of humor rather than its interaction with educational contexts. As a result, some scholars advocate for categorizing instructional humor based on its functional role in teaching and learning. Kaplan and Pascoe (1977) categorized instructional humor into three types based on its ability to provide examples for specific concepts: concept humor, non-concept humor, and mixed humor (a combination of the first two types). Similarly, Vance (1987) identified two types of humor: contiguous humor, which serves to connect the instructional process and typically occurs before or after a knowledge segment, and integrated humor, designed to elucidate knowledge and interspersed within the content. Wei et al. (2010) further refined the functional distinction of humor in education through the observation and analysis of teaching competitions, identifying 23 specific humor techniques within five categories: humor responding to societal issues (e.g., humorous comments on news), humor elucidating knowledge (e.g., amusing illustrations of concepts), humor related to history (comments on historical events and figures), humor involving the teacher (humorously answering their own questions), and humor addressing students (responding to student queries humorously). This study broadens the understanding of instructional humor’s role beyond mere knowledge illustration, although its classification, based on limited samples, may not encompass the full spectrum of humor used in classrooms. Tamblyn (2003) not only considered the role of humor in elucidating knowledge but also its impact on the teacher-student relationship and expanded the variety of instructional humor to include concept humor, which aids in understanding knowledge; friendly humor, which strengthens interpersonal connections; hostility humor, which elevates the teacher’s status but may generate a negative atmosphere; self-disparaging humor, which entertains others; and neutral humor, which serves no specific function related to people or knowledge. This approach highlights the complex nature of humor in education, recognizing its potential to influence both cognitive and relational dynamics in the classroom.

Subsequent research has increasingly acknowledged the unique role of humor in educational settings, emphasizing the need for classifications that account for its appropriateness and impact on students. Martin et al. (2003) proposed a two-dimensional framework for humor based on its target (self or others) and its consequences (beneficial or harmful). Wanzer et al. (2006) suggested that instructional humor should be divided into two general categories: appropriate and inappropriate, considering the outcomes of humor usage in classrooms. They analyzed student recollections of their teachers’ humor and identified four types of appropriate humor containing 26 subtypes, as well as four types of inappropriate humor consisting of 27 subtypes based on their answers. This categorization takes into account the target, function, and effects of instructional humor, highlighting that humor related to class material, unrelated class material humor, self-deprecating humor, and unintentional humor are generally deemed appropriate, with the first two types being most commonly employed in classrooms. Conversely, inappropriate humor includes student-targeted disparaging humor, other-targeted disparaging humor, self-deprecating humor, and offensive humor (sexual and vulgar), with the first category being notably prevalent among inappropriate humor instances. This suggests that humor disparaging individuals, regardless of the target, is often perceived as inappropriate by students. For instance, students perceive self-deprecating humor that labels oneself as an “idiot” as inappropriate, whereas they welcome the sharing of embarrassing personal anecdotes (Wanzer et al., 2006). Thus, the key concerns for students in terms of humor appropriateness are aggression and derogation. Frymier et al. (2008) refined the categorization of instructional humor by addressing the overlaps identified in Wanzer et al.’s (2006) work, ultimately presenting five distinct categories through factor analysis: related humor, unrelated humor, other-disparaging humor, self-disparaging humor, and offensive humor. They intentionally excluded unintentional humor, noting that such instances could be accommodated within the other defined categories. Their findings underscored related humor as the most fitting and beneficial for teaching and learning while categorizing other-disparaging humor as the least appropriate for educational settings. Building upon this framework, Bieg and Dresel (2016) proposed a similar classification system, delineating instructional humor into four main types: humor related to course material, humor unrelated to course material, self-disparaging humor, and aggressive humor. The latter category encompasses both other-disparaging and offensive humor, highlighting these as fundamentally anti-social behaviors that are likely to cause harm. This nuanced approach to categorizing instructional humor has been embraced in subsequent research, celebrated for its clarity and practical applicability in educational contexts (Bieg et al., 2019; Gao et al., 2022; Luo and Zhan, 2021; Tsukawaki and Imura, 2019). Additionally, Tunnisa et al. (2019) introduced two novel humor types—unresponded humor and remind humor—identified through qualitative analysis in a small-scale English course. Despite offering new insights, the limited scope of their study suggests a need for further research to validate these findings across broader educational settings.

To summarize, the classification of instructional humor varies significantly due to its complex nature. However, to understand how humor interacts with education effectively, a clear classification method that considers both appropriateness and inclusiveness is essential. As a result, researchers have largely agreed upon categorizing instructional humor into four or five main types, as proposed by Bieg and Dresel (2016) and Frymier et al. (2008), respectively. These categories include related humor, unrelated humor, and self-disparaging humor, which are considered appropriate and beneficial in educational contexts (Table 1). These types of humor, especially related and unrelated humor, have been the focus of many studies exploring their impact on teaching and learning.

**Table 1:** Appropriate types of instructional humor in educational settings.

Type	Feature	Target	Function	Method
Related humor	Related to class materials or course contents	Knowledge	Illustrate concepts, amuse students, etc.	Tell jokes related to course contents, use funny props to illustrate concepts, etc.
Unrelated humor	Unrelated to class materials and course contents	Others (except knowledge, students, and other specific people)	Amuse students, relieve tension, etc.	Tell stories unrelated to course contents, use sarcastic humor about general topics unrelated to the course, etc.
Self-disparaging humor	Related to teachers’ self-experiences	Teachers	Elicit social emotion, build solidarity, etc.	Make fun of themselves in class, tell embarrassing stories about themselves, etc.

### 3.3 Theories of instructional humor

The theoretical framework elucidating the function of instructional humor in teaching and learning processes is based on three principal kinds of humor theories that elucidate the creation and purpose of humor and why it is effective. The first theory, superiority (or disparagement) theory, posits that humor originates from a sense of superiority felt when comparing oneself favorably against those deemed inferior or unfortunate (Wicker et al., 1980). Likewise, Zillmann and Cantor (1996) proposed disposition theory and asserted that humor is more likely to elicit a positive emotional response when the subject of the humor is neither someone the audience cares for nor a group with which they identify (Zillmann and Cantor, 1996). Secondly, the arousal theory (Berlyne, 1969) and the relief theory (Freud, 1976) defines humor as a pleasurable experience (i.e., mirth) triggered by a mix of cognitive assessment and physiological arousal, aiming to alleviate depression and tension through laughter. Thirdly and the most importantly, incongruity-resolution theory, emphasizes that humor is generated by the recognition incongruous information and the resolution of this contradiction, highlighting on the cognitive understanding of humor rather than its social and emotional functions (Suls, 1983; Suls, 1972).

While the previously mentioned theories of humor address why humor elicits laughter and how it is cognitively processed, their application to the impact of humor in educational settings, particularly on learning outcomes, remains unclear. To bridge this gap, researchers have begun to establish a theoretical framework for instructional humor, drawing on existing humor theories to elucidate the beneficial effects of humor-infused teaching methods on learning. Frymier et al. (2008) highlighted two critical criteria for evaluating the use of humor in the classroom, informed by disposition and incongruity-resolution theories: appropriateness and effectiveness. Specifically, instructional humor is likely to be perceived positively by students when it does not target individuals or groups they are closely affiliated with, and when any incongruity presented is effectively resolved. In contrast, humor that disparages or creates a negative classroom atmosphere is deemed inappropriate for instruction, as it contradicts students’ expectations for the class environment and may hinder their ability to process and resolve incongruous information subsequently. However, despite these insights, the existing literature does not fully elucidate why instructional humor enhances learning.

#### 3.3.1 Instructional humor processing theory

The Instructional Humor Processing Theory (IHPT), proposed by Wanzer et al. (2010), firstly offers an integrative framework for understanding how students process and interpret humorous content in educational contexts. This theory incorporates elements from incongruity-resolution theory, disposition theory, and the Elaboration Likelihood Model of persuasion (ELM) to explain the cognitive mechanisms behind students’ responses to instructional humor. IHPT is predicated on the essential role of incongruity recognition and resolution: students must first identify the incongruity within the humorous material and then resolve it. Failure to do so may confuse, especially if the incongruity presented is overly absurd or complex. IHPT underscores a critical precondition for the effective use of humor in educational settings—the students’ successful identification and understanding of humor. Once the humorous content is acknowledged, students then assess its humorousness based on the target of the humor. Consistent with disposition theory, students are more inclined to view humor positively and accept it when it is directed at individuals or groups with whom they do not identify or favor. In summary, the effective perception and acceptance of humor, both cognitively and emotionally, lead to laughter, increased attention, and enhanced motivation toward the course material.

Most importantly, IHPT further elucidates the impact of instructional humor on learning by integrating ELM, which outlines two distinct routes of persuasion: central and peripheral (Cacioppo and Petty, 1984; Petty and Cacioppo, 1986). ELM posits that in peripheral route processing, individuals focus on the superficial elements of information, leading to minimal changes in cognitive structures. In contrast, central route processing engages individuals more deeply with the content’s arguments, facilitating substantial elaboration and personal integration of the information. IHPT emphasizes the critical role of humor that is directly related to course material, such as humorous examples or illustrations of concepts. This approach to instructional humor has a dual effect. Firstly, it captures students’ attention through its inherent entertainment value, creating a cognitive dissonance due to the incongruity with their existing beliefs and knowledge frameworks. This dissonance, combined with an increased motivation prompted by the humor, leads to enhanced processing and recall of the information. Secondly, the additional context provided by humorously presented content aids in clarifying and solidifying the understanding of knowledge, improving students’ comprehension. Thus, the application of humor in teaching, particularly when it is relevant to the course content, not only maintains student attentional engagement but also fosters an environment conducive to increased motivation and a deeper capacity for knowledge processing (Wanzer et al., 2010).

**Figure 1:**
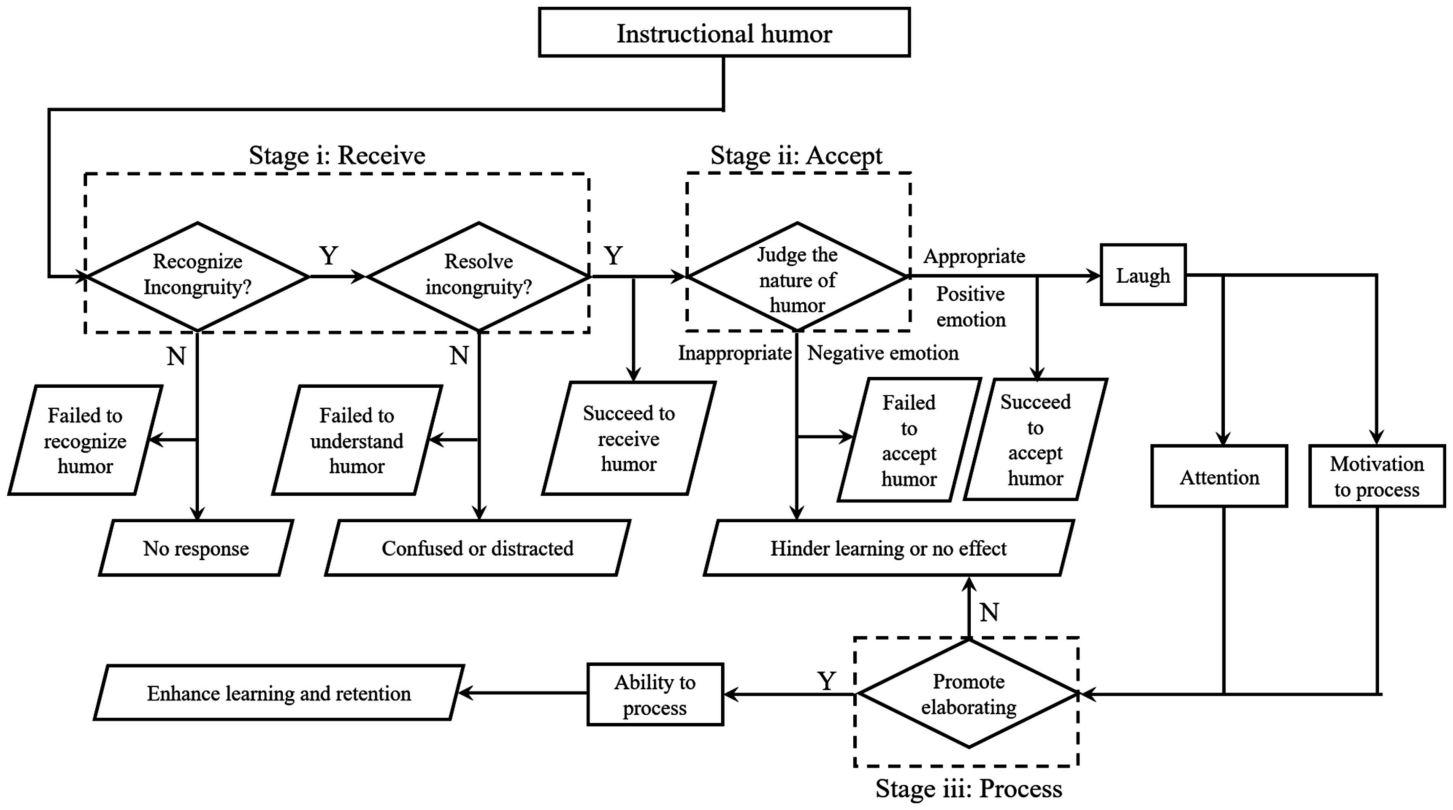
The explanation from IHPT of how instructional humor is processed by students and affects learning.

In summary, IHPT proposes a three-step procedure through which instructional humor influences teaching and learning from the perspective of the recipient, encompassing reception, acceptance, and processing of humorous content (Figure 1). IHPT reveals that only being humorous and entertaining in the classroom is insufficient for enhancing learning outcomes; laughter should not be considered the primary indicator of successful humor integration. Instead, instructional humor’s effectiveness depends on its ability to facilitate students’ engagement with and comprehension of the course material, beyond their initial understanding and approval of the humor. Therefore, IHPT identifies two critical components of instructional humor’s impact on learning. The first component is motivation, suggesting that humor must be deemed appropriate and elicit positive emotional reactions from students to capture their attention and increase their motivation to engage with instructional content. The second component is ability, emphasizing humor’s role in augmenting the elaboration of course content and bolstering students’ comprehension of instructional material. However, it is important to note that the pathways proposed by the IHPT model, detailing the mechanisms through which humor influences learning, were not empirically validated within Wanzer et al.’s (2010) study despite the theory’s significant contribution to the understanding of instructional humor.

#### 3.3.2 Alternative theoretical frameworks of instructional humor

**Figure 2:**
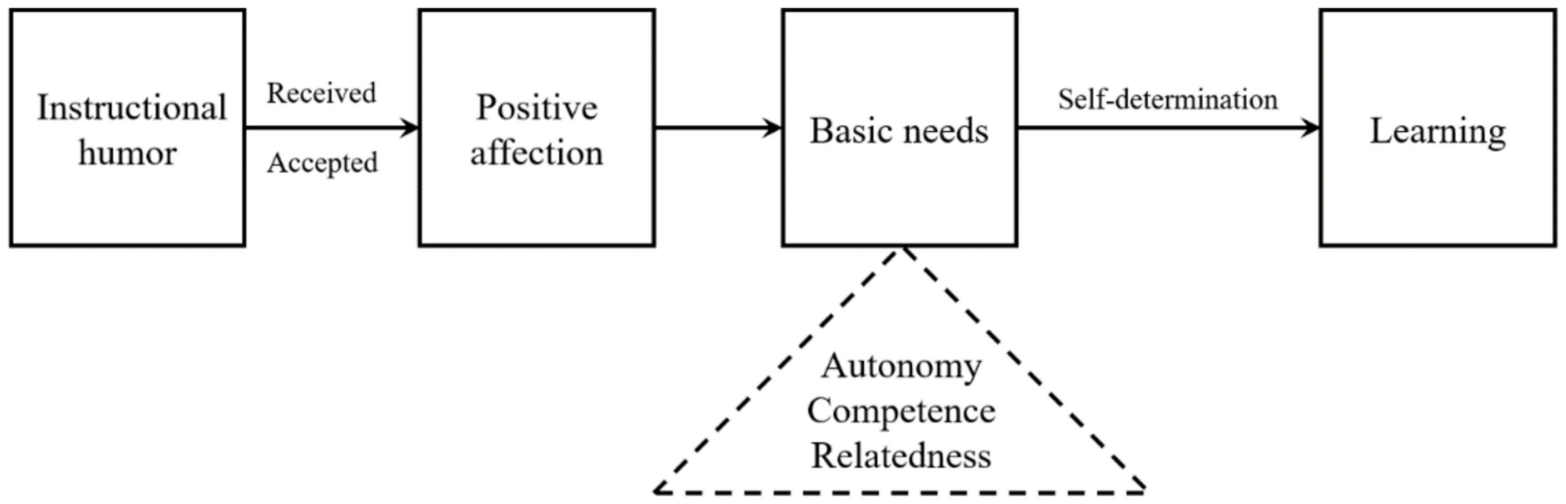
The explanation of how instructional humor affects learning from an alternative theory of IHPT based on SDT.

The absence of empirical data in the IHPT model of Wanzer et al.’s (2010) work spurred further investigation into the relationship between instructional humor and learning. Bolkan and Goodboy (2015) conducted an exploratory study to test the assumptions of IHPT, particularly examining whether there is a direct link between instructional humor, student engagement, and learning outcomes. They evaluated students’ cognitive engagement and sustained attention through self-reports as proxies for the processing ability and attention highlighted in IHPT. Additionally, they assessed student motivation via measures of affective learning based on the premise that positive emotions elicited by humor would encourage deeper engagement with the material. To determine learning achievements, they utilized a perceived cognitive learning scale self-reported by the students. Contrary to IHPT’s predictions, Bolkan and Goodboy found that the model did not fit well; students’ attention, along with their motivation and ability to process information, did not correlate with learning outcomes as IHPT had suggested. Instead, Bolkan and Goodboy turned to Self-Determination Theory (SDT) to explain the benefits of instructional humor. According to SDT, motivation is enhanced when individuals’ innate psychological needs for autonomy, competence, and relatedness are met, leading to self-directed learning and improved performance (Deci and Ryan, 1985; Deci et al., 1991). Their analysis indicated that the primary value of instructional humor in learning might stem from its ability to satisfy these basic psychological needs through positive emotional impacts rather than from increasing attention to the course material. Specifically, appropriate instructional humor was found to enhance students’ perceptions of competence and relatedness with instructors, which, in turn, fostered a greater sense of autonomy in their academic endeavors due to increased self-efficacy and enjoyment of the class (Figure 2). This shift in focus from the cognitive benefits of humor (as proposed by IHPT) to its affective functions (as suggested by SDT) highlights the significant role that the emotional response to humor can play in enhancing learning outcomes. However, the reliance on self-reported measures of cognitive engagement and affective learning in Bolkan and Goodboy’s study raises questions about the accuracy of these proxies in capturing the essence of IHPT’s assumptions regarding humor’s role in learning. Additionally, indirectly investigating motivation and learning performance through self-reporting affective learning and perceived cognitive learning is insufficient to validate or refute the IHPT theory, thereby calling for further study to assess the elements of IHPT in a more direct way.

**Figure 3:**
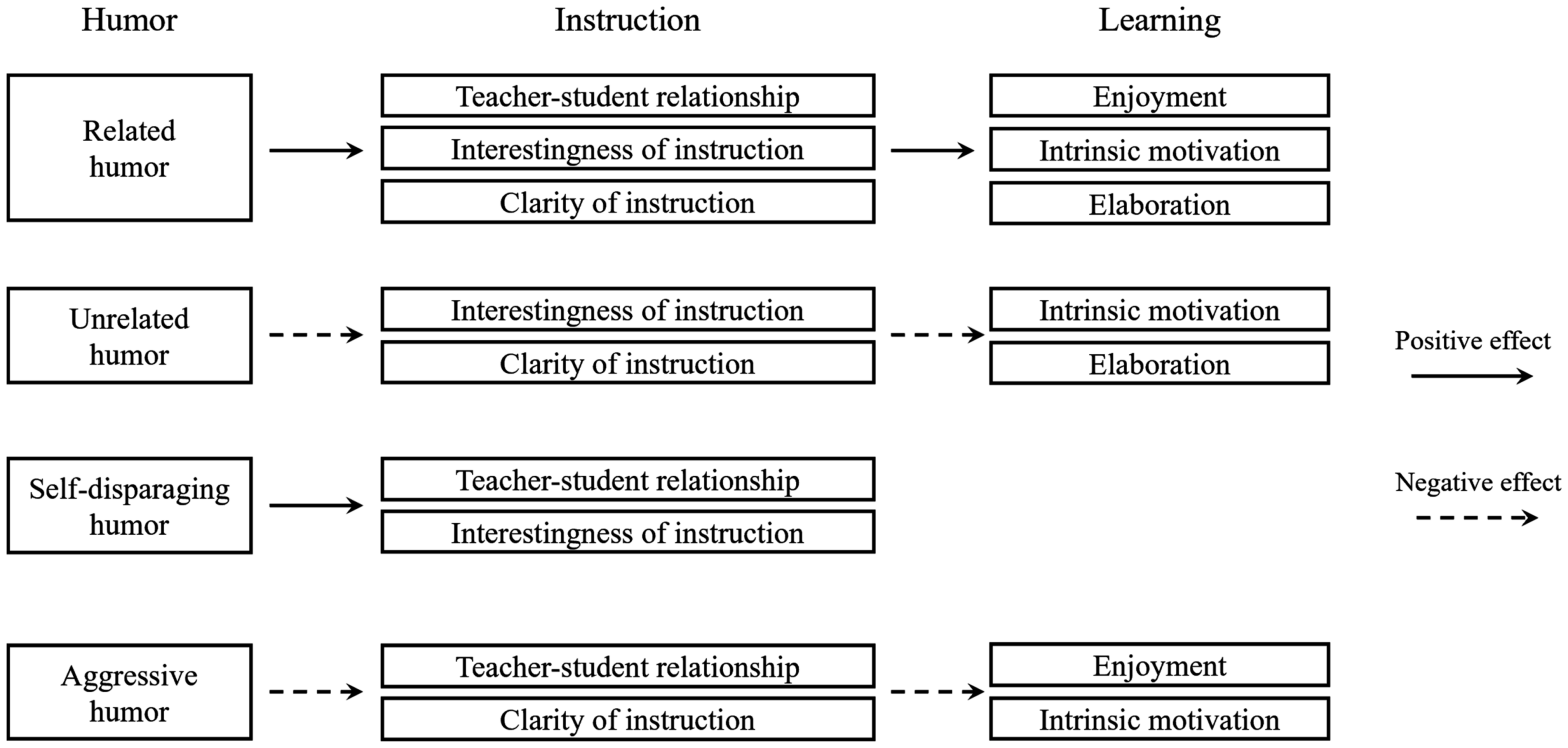
The explanation of how instructional humor affects learning from an integrative perspective of cognition and affection.

As mentioned before, Wanzer et al. (2010) highlighted the benefits of instructional humor from the perspective of information processing, whereas Bolkan and Goodboy (2015) emphasized the affective advantages of humor. Bieg and Dresel’s (2018) study proposes a comprehensive model that evaluates both the cognitive and affective roles of various types of instructional humor on students’ learning outcomes. The study posits that class-appropriate humor not only enhances social emotions and motivation but also aids in garnering attention and elucidating content, reflecting humor’s dual impact on affective and cognitive domains. Survey findings suggest that humor related to the subject matter positively influences learning by engaging both affective and cognitive aspects of instruction. In contrast, aggressive humor detrimentally impacts learning by discouraging affiliation and obstructing the processing of instructional content. Surprisingly, humor unrelated to the instruction is also detrimental, potentially because such humor creates confusion. Additionally, while self-disparaging humor does not impede learning, it does not contribute to it either, despite its positive social and emotional impacts on the instructional process. This investigation recognizes the complex nature of instructional humor, indicating that its effects on teaching and learning vary with the type of humor employed (Figure 3). However, unlike IHPT, this model does not detail the mechanisms through which instructional humor influences learning. Moreover, the factors identified as influencing learning outcomes partially overlap with the basic psychological needs outlined in Bolkan and Goodboy’s (2015) model.

In summary, these reviewed models provide theoretical frameworks for understanding how instructional humor influences learning, focusing on cognitive and affective aspects from the students’ perspective. One perspective emphasizes the cognitive benefits of instructional humor, such as increased attention and deeper information processing, while the other highlights its affective benefits, including enhanced motivation and improved interpersonal relationships, leading to positive social emotions in the classroom. The diversity in theoretical orientation contributes to varied empirical research themes within this domain. However, these models face challenges related to inconsistencies and a lack of integration due to differences in underlying concepts, theories, and measurement approaches. This situation calls for further research aimed at elucidating the mechanisms of instructional humor in a more comprehensive manner, with the goal of developing an integrated theoretical model.

## 4 The empirical evidence of instructional humor in class

The authority of teacher advocated by traditional education in the past makes humor almost impossible to happen in class (Davies, 2003). Given that the equality status between teachers and students in today’s new era, instructional humor provides a new possibility for educational practice. Researchers agree that instructional humor is beneficial in instruction on account of its multiple function including increasing social relationship between teachers and students, improving clarity of instructional information, and promoting retention and mastery of knowledge (Al-Duleimi and Aziz, 2016). Instruction is a bilateral activity in which teachers and students engage in the whole process, exactly as the humor attempts requires the usage from productor and the appreciation and understanding from recipient (Mcghee, 1979). The empirical studies regarding to instructional humor are conducted from two perspectives including the teachers’ use of humor and its effects on students.

### 4.1 The use of instructional humor in teaching for teachers

Mikhail Svetlanov, a noted Soviet educator, emphasized that humor serves as a crucial tool for educators. Instructional humor is recognized as an effective strategy for teachers to clarify teaching material and address students’ emotional needs in the classroom (Allen, 2014). When properly utilized, humor in the classroom can motivate students and enhance their learning experiences (Toroka et al., 2004). Therefore, researchers examined the use of instructional humor from the perspective of educators, with the goal of optimizing its effectiveness in teaching environments.

#### 4.1.1 The frequency of instructional humor in class

Two objectives have largely driven existing research on the frequency of humor usage in the classroom. Firstly, similar to ensuring the types of humor used by teachers are appropriate for the classroom environment, there is a focus on identifying the optimal frequency of humor attempts during class sessions. Ziv (1988) posited that employing humor three to four times per class session could be an ideal balance, arguing that excessive humor can reduce its beneficial impact on teaching and learning. This perspective was empirically tested in a semester-long course where three jokes, each elucidating course concepts, were incorporated into each class, resulting in improved student performance on the final exam. Similarly, Masek et al. (2019) incorporated humor at least three times per class over a 14-week course, observing that students reported a positive perception of their teachers’ use of humor and demonstrated enhanced engagement. These studies offer initial evidence supporting the notion that there is an appropriate frequency for the use of instructional humor within classroom settings.

Another aspect of research on humor frequency is the investigation regarding the question of how often teachers use humor in instruction. In the past, teachers used humor approximately once every 15 min (i.e., 3.34 times per class), according to a random analysis of college classrooms (Bryant et al., 1979). The frequency of humor usage among teachers varied significantly, with one in five teachers not using humor at all, and about 30 % attempting humor four or more times per class, sometimes exceeding 10 times. Nearly half of the teachers used humor between one and three times. Gorham and Christophel (1990) reported a similar finding, with the average number of humor attempts per class ranging between one and two. Recent studies indicate a shift in the frequency of humor usage. Petraki et al. (2016) conducted a qualitative study in university English classes and found that teachers used humor seven to eight times per class, primarily at the beginning and end. Downs et al. (1988) observed an even higher frequency, with humor being used about 13 times in a 50-min college class. Notably, award-winning teachers were found to use humor about seven times per class, which is half as much as their counterparts. Javidi and Long (1989) discovered that experienced teachers used humor approximately 6.5 times per class to elucidate learning content, whereas novice teachers averaged about 1.6 humor attempts, often irrelevant to the material. Javidi et al. (1988) found that secondary school teachers made between two and three humor attempts per class, fewer than college teachers who used humor 7.2 times on average. Neuliep (1991) reported that high school teachers used humor about two times per class, according to self-reports. Furthermore, Shoda and Yamanaka (2022) argued that humor is more prevalent in online classes than in physical classrooms. This was based on an analysis of 50 of the most and least popular TED presentations, which lasted about 15–20 min and featured approximately 13 humor attempts (as indicated by audience laughter) in popular videos, compared to four times in the less popular ones.

The varied findings regarding the frequency of instructional humor suggest several implications. First, the application of humor in teaching might differ according to the audience, with seasoned educators likely having a better grasp of effective humor usage, which supports the notion that adept use of instructional humor constitutes a professional skill that educators can develop over time (Kunter et al., 2013). Second, the preferred frequency of humor has evolved over the years, potentially due to the emergence of new instructional methods. The increase from once every 15 min to approximately once every 1.5 min, as observed by Shoda and Yamanaka (2022), especially in non-traditional classroom settings, highlights this shift. However, this observation, particularly in contexts like TED talks where humor may be employed deliberately to engage the audience, necessitates further verification in authentic educational settings, including online video-based courses. Third, the nature of the course content itself can influence the incorporation of humor, with certain subjects offering more opportunities for humor integration than others (Petraki et al., 2016). Thus, future research could aim to investigate the frequency of humor across a broader spectrum of disciplines and instructional formats. Lastly, there is a pressing need for more comparative studies examining the effects of varying humor frequencies to obtain definitive conclusions about its optimal use in educational contexts. As such, current findings should be interpreted with caution, and the exploration of humor’s effective frequency in education should continue to be supported by additional evidence.

#### 4.1.2 Other factors affecting teachers’ use of instructional humor

The instructional dynamic between teachers and students plays a crucial role in educational processes (Griffiths and Soruç, 2021). Thus, teachers’ personalities and students’ feedback can significantly impact the utilization of humor in the classroom (Petraki et al., 2016). Recognizing the importance of aligning humor with the personalities of both teachers and students, researchers have dedicated considerable effort to understanding this interplay and have derived numerous insights.

##### 4.1.2.1 Teachers-related factors

The utilization of humor in the classroom is significantly influenced by the teacher’s gender, with female teachers generally using humor less frequently than males, particularly in smaller classes (Crawford and MacLeod, 1990). Additionally, a study of 70 college teachers indicated gender differences in the types of humor used (Bryant et al., 1979). Male teachers were found to prefer unrelated humor, such as jokes, humorous anecdotes and self-disparaging humor, while female teachers were more inclined to use spontaneous humor to elucidate instructional content, suggesting that male and female teachers may employ humor with different objectives in mind: men may use it deliberately to enliven the classroom atmosphere and entertain students, whereas women may use humor as a teaching aid, resorting to it only when it serves a pedagogical purpose (Sev'er and Ungar, 1997). The perception and attitudes of students toward their teachers’ humor attempts also reflect gender biases. Humor from male teachers is often seen as a means to engage students, but humorous female teachers can be perceived as less appealing unless they employ aggressive humor (Bryant et al., 1980). Conversely, Darling and Civikly (1987) found that students responded more defensively and perceived humor attempts more negatively when female teachers used tendentious humor, which disparages or offends others, in contrast to male teachers whose use of non-tendentious humor was perceived as less genuine. This discrepancy is thought to arise from gender stereotypes, with men associated with aggression and dominance and women expected to be friendly and supportive. As such, humor that deviates from these gender expectations can be seen as a self-protective measure rather than an instructional strategy (Darling and Civikly, 1987). However, Banas et al. (2011) reported that the gender difference in humor usage observed in studies had a small effect, aligning somewhat with Van Giffen’s (1990) finding that gender stereotypes in humor usage diminish when students feel a sense of familiarity and closeness with their teachers, which supports the idea that teachers can effectively use humor and be positively received by their students when perceived as trustworthy, approachable, and professional (Wrench and Richmond, 2004). The varied perspectives on the influence of gender on humor in the classroom indicate the need for further research, particularly studies that examine the interaction between gender differences in humor usage and the teacher-student relationship.

When considering teachers’ humor usage in class, the second important factor influencing humor is humor orientation, which refers to the ability to produce humor and be funny (Booth-Butterfield and Booth-Butterfield, 1991). Teachers with a high humor orientation have increased social skills, enabling them to use humor as a tool to foster closer relationships with students and enhance their satisfaction (Wanzer and Frymier, 1999). This is attributed to the perception of students who view teachers capable of effectively employing humor as competent and professional, as humor usage is often associated with confidence and responsiveness (Wanzer, 1995). Thus, teachers with a high humor orientation are more inclined to utilize humor more in classrooms compared to those with a lower humor orientation (Frymier et al., 2008). In addition, Wanzer et al. (2010) observed that teachers adept in humor are sometimes willing to use even inappropriate forms of humor, such as other disparaging humor, to foster an entertaining classroom atmosphere due to their proficiency in humor execution and a more complex understanding of humor dynamics. In essence, teachers with a strong humor orientation are more effective in leveraging humor to diminish psychological barriers with students and engage them more effectively in the educational process, thereby increasing their satisfaction with the course (Aylor and Opplinger, 2003).

Other individual differences among teachers also influence their use of humor in instruction. For instance, teachers with extensive experience or those who have received awards typically employ instructional humor to clarify messages and illustrate content more effectively (Downs et al., 1988; Javidi and Long, 1989; Javidi et al., 1988). Daumiller et al. (2019) reached a similar conclusion, noting that seasoned teachers tend to use more related humor compared to their less experienced counterparts. Their research also explored how teachers’ achievement goals impact their humor use, finding that teachers with performance-avoidance goals are less likely to incorporate humor into their teaching. This reluctance is attributed to the fear that humor might be perceived negatively by students, potentially threatening the teacher’s self-worth. Furthermore, Gorham and Christophel (1990) discovered that teachers with low immediacy are more inclined to use tendentious humor than those with high immediacy, as their students are less likely to view their inappropriate humor as offensive. They advised that teachers lacking immediacy should ensure their humor is relevant to the content to avoid being perceived as digressive.

##### 4.1.2.2 Students-related factors

Student-related factors also play an important role in the effectiveness of humor use in the classroom. Educators need to consider students’ gender when incorporating humor into their teaching strategies. The perception of humor varies between male and female students, mirroring the observed differences in humor usage among male and female teachers. Cooper et al. (2018) examined 25 college courses and observed that jokes made by teachers were more likely to offend female students, whereas male students were more inclined to find them amusing. This discrepancy may be attributed to the neural differences in humor processing identified by Kohn et al. (2011), where men typically engage in a comprehensive cognitive evaluation of the humor’s connotation and function, while women tend to concentrate on the emotional aspects of humor, due to heightened activity in the emotional processing areas of the brain, leading to their aversion to certain types of humor. Contrastingly, other studies (Chen, 2017; Machlev and Karlin, 2017; Machlev and Karlin, 2016) present an alternative viewpoint, suggesting that male and female students share similar perceptions and preferences toward appropriately executed humor in the classroom, which indicates that the impact of a teacher’s use of humor may not significantly vary based on the gender of the students, provided the humor is used appropriately, such as selecting suitable types of humor. However, this hypothesis highlights the need for further research to reconcile these conflicting findings and to explore how different genders perceive humor attempts when executed properly by educators.

Beyond the inherent differences in gender, researchers have investigated additional factors among students that could influence the effectiveness of teachers’ humor in the classroom. One such factor is the students’ sense of humor, which shapes their response to teachers’ humor. Lu et al. (2023) discovered that students with a high sense of humor were more engaged in class following humor attempts by teachers, attributed to their enhanced capacity to understand humor. Furthermore, the variation in students’ knowledge and skills also plays a role in how humor is perceived, especially in the context of foreign language learning. Non-native speakers may struggle to grasp humorous content due to limited language proficiency and unfamiliarity with the cultural context (Chen, 2017), whereas students with higher language skills are more likely to appreciate and accept the humor used by their teachers (Neff and Rucynski, 2017). Students’ preferences for certain humor strategies in foreign language classes also correlate with their enjoyment of the language and their attitudes toward instructional humor (Neff and Dewaele, 2022). Furthermore, Praag et al. (2017) emphasized the importance of paying attention to the difference in students’ social backgrounds for teachers when employing instructional humor in class. They found that students from academic backgrounds preferred humor related to course content and devoid of malice, while those from vocational or technical backgrounds favored aggressive humor targeting specific subjects, interpreting it as an attempt by teachers to establish a closer relationship rather than to offend. This finding emphasizes that instructional humor can avoid misunderstanding and conflict when there is alignment between the backgrounds and humor perceptions of teachers and students. Similarly, Wu et al. (2023) suggested that teachers should use humor matching the humor styles of their students. They conducted surveys with nearly a 1,000 students, asserting that students are more likely to respond positively and feel a sense of closeness to teachers’ humor when it aligns with their own humor preferences and when teachers use humor styles that students favor. Finally, cultural background is a potential factor influencing the employment of instructional humor in class. For instance, humor is not valued in Chinese classrooms because it is seen as an avoidance behavior in coping with problems and stress in traditional Chinese culture (Wu and Chan, 2013). Influenced by Confucian culture, Chinese individuals often perceive humor as vulgar and inappropriate (Yue, 2010). Thus, Chinese students may view teachers who utilize humor as lacking maturity and competence rather than being wise and capable. Furthermore, the use of humor by teachers could exacerbate communication apprehension among Chinese students, as humor highlights individuality within a collective setting, thereby increasing pressure in China’s collectivist culture (Zhang, 2005). Therefore, educators should tailor their use of instructional humor to accommodate the diverse backgrounds of their students rather than assuming its universal effectiveness. Nevertheless, research in this area remains scarce and needs to be further investigated.

### 4.2 The effects of instructional humor on learning for students

The utilization of instructional humor in educational settings aims to engage students and improve their learning by making the process more enjoyable (Sahin, 2021). As a result, the impact of humor on learning has garnered considerable attention from scholars in the field. This interest has been bifurcated into two main research directions due to the differing views regarding the rationales of instructional humor, focusing on cognitive and non-cognitive outcomes of humor in education. Researchers advocating for the cognitive benefits of humor concentrate on its influence on students’ cognitive performance. This perspective is grounded in the understanding that humor, closely linked to emotion, can enhance the memorability of information and its retention in long-term memory due to the affective dimensions of our experiences (Lujan and Dicarlo, 2016). Conversely, studies emphasizing the non-cognitive aspects value humor for its role in fostering a positive learning atmosphere. This approach posits that instructional humor primarily boosts student engagement rather than directly facilitating access to educational content (Bolkan et al., 2018). As a result, these studies focus more on the effects of instructional humor on affective performance.

#### 4.2.1 Cognitive learning performance

Building on the framework proposed by Wanzer et al. (2010), which introduced IHPT highlighting humor’s role in enhancing the processing of instructional information, numerous scholars have reported the cognitive benefits of humor in learning environments. The underlying principle is that positive emotions elicited by humor can enhance students’ elaboration processes, ultimately improving their learning outcomes (Stark et al., 2018), attributed to the laughter and enjoyment experienced in response to humor, especially when presented in a serious context, making the humorous content more memorable than its non-humorous counterparts (Purzycki, 2010). Research on the cognitive impact of humor on learning has primarily focused on two areas. The first and most extensively studied is learning achievement. This area considers how humor assists in the acquisition and recall of knowledge, viewing learning achievement as a direct indicator of the efficacy of humor in educational settings. Additionally, the engagement in vigorous cognitive activities required for processing concepts is considered a fundamental prerequisite for achieving significant learning outcomes (Parong and Mayer, 2021). Consequently, numerous studies have assessed the complex cognitive processes experienced by students during learning, aiming to elucidate the mechanisms through which humor contributes to observable learning achievements.

##### 4.2.1.1 Learning achievement

The relationship between instructional humor and learning achievements has garnered extensive empirical attention, yielding mixed results. A significant portion of the research community supports the notion that instructional humor enhances learning, as evidenced by studies focusing on perceived cognitive learning. Perceived learning refers to students’ self-assessment and anticipation of their cognitive learning performance (Bolinger and Stanton, 2014). Bolkan and Goodboy (2015) observed that instructional humor positively influences perceived learning outcomes, with students reporting higher satisfaction with their learning achievements and a better grasp of knowledge when they recognize teachers’ efforts to incorporate humor into lessons. This is attributed to the role of instructional humor in signaling a teacher’s intention to make the learning experience more engaging and to clarify concepts in an enjoyable manner (Dresel et al., 2014). Existing research indicates that the impact of instructional humor on perceived cognitive learning varies according to the type of humor employed, echoing previous categorizations. Related humor, which is pertinent to the course content, is consistently viewed as beneficial to students’ self-reported cognitive learning achievements. However, the effects of unrelated humor and self-disparaging humor on learning are more variable (Masek et al., 2019; Tsukawaki and Imura, 2019; Wanzer et al., 2010). Bieg and Dresel (2018) further argued that humor unrelated to course content can obstruct students’ perception of learning by not aiding in the elaboration or clarification of knowledge, potentially causing confusion. Conversely, inappropriate humor, particularly when it is tendentious or disparages students, is shown to negatively affect perceived cognitive learning outcomes (Gorham and Christophel, 1990).

However, it is important to distinguish between students’ self-perception of learning and their actual knowledge acquisition (Horzum et al., 2015). Machlev and Karlin (2016) demonstrated that while appropriate humor in lessons can enhance students’ perceived learning performance, it does not necessarily translate to actual academic achievements and grades. Perceived learning is influenced by students’ self-assessment and beliefs (Caspi and Blau, 2008), whereas actual learning performance reflects changes in cognitive structure resulting from the instructional process (Soderstrom and Bjork, 2015). Consequently, some researchers favor using objective test scores as a measure of instructional humor’s effectiveness, arguing that it more accurately represents students’ knowledge improvement. It is established that humor designed within lessons aids immediate memory and recall of instructional content (Chapman and Crompton, 1978; Vance, 1987) and is particularly beneficial for students with lower levels of creativity (Clabby, 1979). Hackathorn et al. (2012) explored instructional humor’s impact on cognitive learning outcomes through Bloom’s taxonomy, finding that humor use in classes boosted exam achievements, particularly in knowledge and comprehension, rather than application. This positive impact of instructional humor is not limited to traditional classroom settings but extends to online education, where students achieve satisfactory outcomes when humor is integrated (Wegener, 2022). Moreover, the effectiveness of instructional humor varies by type. Related humor, which is connected to course content, has been shown to significantly enhance students’ actual cognitive learning performance (Allen, 2014; Celik and Gundogdu, 2016; Ziv, 1988). However, empirical evidence supporting the advantages of the other two appropriate humor types is scarce. Wang and Chen (2022) found that humorous videos unrelated to course content could improve learning transfer, albeit without enhancing knowledge retention. Luo and Zhan (2021) reported that teachers’ use of all types of humor in online instruction, including related, unrelated, and even aggressive humor, could positively predict students’ academic scores from the previous semester. This finding, while intriguing, should be approached with caution and requires further validation, as it challenges prevailing theories on the impact of inappropriate humor on learning.

Despite the documented advantages of instructional humor, several studies present less favorable views on its positive impacts on cognitive learning outcomes. Both in offline and online educational settings, students do not demonstrate enhanced knowledge acquisition or improved performance due to instructional humor, even though they report increased motivation to learn (Conkell et al., 1999; Houser et al., 2007). Furthermore, humorous illustrations in textbooks do not contribute to the student’s acquisition of information (Bryant et al., 1981). Some researchers have raised concerns about the potential negative effects of instructional humor, arguing that attempts to integrate humor into courses may impede learning (Bolkan et al., 2018; Weaver et al., 1988). These studies have observed that students learning with humorous materials exhibit poorer performance in retention and transfer tests compared to those who learn without humor. Moreover, these students struggle to grasp knowledge and concepts conveyed through humor, showing a tendency instead to remember the humorous content itself more effectively.

The mixed findings regarding the impact of instructional humor on cognitive learning outcomes may arise from its effectiveness being more aligned with long-term processes rather than short-term exposure. Ziv (1988) was among the first to conduct a longitudinal study over a semester, integrating related humor (i.e., three jokes to illustrate concepts) into each lesson. The results showed that students achieved higher academic scores when learning with instructional humor compared to a non-humorous approach, a finding that was replicated in another semester-long course taught by a different professor. Similarly, employing related humor, such as using humorous examples and cartoons to illustrate concepts, was found to enhance retention and recall of knowledge in tests conducted 6 weeks post-instruction, even if the immediate effects of humor were not evident (Celik and Gundogdu, 2016; Kaplan and Pascoe, 1977). Contrarily, a longitudinal study by Machlev and Karlin (2016) revealed that related humor in class did not correlate with students’ actual learning achievements as measured by their final scores. This suggested that despite being motivated by the instructional humor over time, students did not in fact acquire additional knowledge.

##### 4.2.1.2 Cognitive activity during learning

The IHPT posits that the critical cognitive function of instructional humor in enhancing learning is its capacity to boost attention and facilitate deeper elaboration of material (Wanzer et al., 2010). Research in this area has primarily investigated instructional humor’s effectiveness in capturing and sustaining attention by self-report questionnaires. There is a consensus among educators and learners that appropriately deployed humor in the classroom can significantly enhance attention and maintain student focus on the course content, provided the humor is engaging (Bakar and Kumar, 2019; Cooper et al., 2018). For instance, it has been observed that young children show increased concentration on instructional content and maintain interest in educational TV programs that incorporate humor (Zillmann et al., 1980). However, there are concerns that instructional humor might divert attention from the learning material to the humorous elements, potentially impairing educational outcomes due to the amusing nature of humor (Bolkan et al., 2018; Weaver et al., 1988). The discrepancy in findings across studies often relates to the types of humor used in the classroom. Humor that is related to the course material has been shown to effectively direct student attention toward the content, thereby aiding in learning achievement (Bieg and Dresel, 2018; Tsukawaki and Imura, 2019). This was corroborated by a longitudinal study by Masek et al. (2019), which demonstrated that employing humor relevant to the course content over 14 weeks resulted in enhanced student engagement and sustained attention, as indicated by assessments at the semester’s end. Conversely, humor that is unrelated to the subject matter may detract from students’ focus on the course and diminish their interest, as the amusement derived from the humorous elements can overshadow the educational content (Machlev and Karlin, 2017). Such overuse of unrelated humor is often perceived by students as a distraction, negatively impacting their perception of the instructional approach (Bieg et al., 2017).

The second critical aspect of study within this domain focuses on elaboration, a concept central to IHPT, which posits that the primary goal of employing humor in educational settings is to enhance students’ processing and comprehension of the material (Wanzer et al., 2010). Savage et al. (2017) suggested that by capturing attention, humor facilitates deeper engagement with instructional content, leading to improved understanding. Research exploring the impact of instructional humor on elaboration has primarily used self-report questionnaires to assess students’ perceptions of their processing abilities. Findings indicate that students report enhanced comprehension and ability to elaborate on knowledge when instructors use humor related to the course content (Bieg and Dresel, 2018; Tsukawaki and Imura, 2019). These results align with IHPT’s viewpoint that humor, particularly when related to the instructional content, is effective in enhancing the processing of information, which is attributed to the creation of conceptual links between the humorous elements and the learning material, thereby providing additional avenues for understanding (Hu et al., 2017). Contrarily, Suzuki and Heath (2014) reported that instructional humor, even when relevant to the content, did not enhance material elaboration or understanding. Their study showed that students learning through humorous video lectures were more adept at recognizing specific details of the instruction compared to their counterparts exposed to non-humorous content. However, there was no significant improvement in their recall and retention abilities, which are crucial for the elaboration of knowledge. This raises concerns that the emotional engagement elicited by humor may detract from systematic information processing and detailed elaboration, as students may shift their cognitive focus to the entertaining aspects of the content (Pekrun and Linnenbrink-Garcia, 2012). The requirement to resolve the incongruity presented by instructional humor could lead to the allocation of cognitive resources away from encoding the core concepts toward processing the humorous elements. This diversion can create an additional cognitive load and result in a deeper encoding of the humorous content rather than the educational material itself (Frymier et al., 2008; Ruch and Hehl, 2007). For example, Kim and Vishak (2008) demonstrated that the entertainment value of humorous content did not facilitate the processing and acquisition of political information, as readers were more inclined to engage with the content emotionally rather than cognitively, suggesting that while humor can engage students, its effect on actual learning and information processing requires further empirical investigation.

Empirical research to date has examined the connection between instructional humor and student learning performance, focusing on cognitive dimensions. Despite these efforts, several gaps and areas for future research remain evident. One important limitation is the predominant focus on related and unrelated humor, with a notable lack of attention to self-disparaging humor. The cognitive impact of these types of humor, particularly unrelated humor, has not been definitively established. Given that instructors often employ unrelated humor spontaneously and without prior planning (Sahin, 2021), it is crucial to further explore and elucidate the effects of both unrelated and self-disparaging humor. Additionally, distinguishing the cognitive functions of self-disparaging humor from unrelated humor in the learning process is essential, as both are not directly linked to course content. Moreover, there is a pressing need for more empirical evidence, particularly from experimental studies, to support the practical advantages of instructional humor. The majority of existing research relies on surveys, which may not fully capture humor’s actual impact on learning outcomes. Furthermore, the potential long-term benefits of instructional humor necessitate further longitudinal studies to provide concrete evidence. Most critically, there is a lack of research exploring the cognitive activities of students engaged in learning with humor. This gap hinders a deeper understanding of how instructional humor influences cognitive learning processes and outcomes. The existing limited research predominantly relies on self-reported data collected post-learning, which may be subject to biases in students’ subjective assessments. Therefore, a promising direction for future research involves the real-time measurement of students’ cognitive processes during learning experiences through objective and precise instruments, such as eye-tracking, EEG, and fMRI. Such approaches could offer physiological evidence of the cognitive benefits of instructional humor, potentially reconciling the conflicting findings of previous studies.

#### 4.2.2 Affective learning performance

In educational research, numerous studies have shifted focus toward the non-cognitive benefits of instructional humor, particularly its impacts on students, which predominantly centers on the effects of appropriate humor, as students tend to react negatively to humor that they perceive as inappropriate or hostile (Gorham and Christophel, 1990; Nienaber et al., 2019). Such negative reactions stem from the power dynamics in the classroom, where students might feel unable to respond to teachers’ hostile humor, leading to a negative control appraisal and diminished enjoyment in learning (Bieg et al., 2019). Furthermore, inappropriate humor can adversely affect students’ perceptions of a friendly classroom environment, subsequently decreasing their interest and motivation to learn (Bieg and Dresel, 2018; Stuart and Rosenfeld, 1994; Tsukawaki et al., 2020). Although the impact of inappropriate humor—particularly that which is disparaging or offensive—may vary based on the level of aggression in the humor and the relational closeness between teachers and students (Tsukawaki, 2018), it is generally accepted that appropriate humor is affiliative and favored by students. The primary objectives for teachers employing humor include fostering student motivation and participation, creating a relaxed classroom atmosphere, and establishing comfortable teacher-student relationships (Petraki et al., 2016). Consequently, research in this area has largely investigated the affective learning performance associated with the use of appropriate instructional humor, examining its influence from above multiple perspectives.

##### 4.2.2.1 Teacher-student relationship and class atmosphere

Regarding the social dimension, the link between instructional humor and the dynamics of teacher-student relationships, as well as the overall classroom atmosphere, has been a focal point of research. Studies consistently indicate a preference among students for teachers who integrate humor into their instruction, noting that such an approach enhances the closeness of teacher-student relationships and promotes social interaction (Abraham et al., 2014; Aylor and Opplinger, 2003; Chiarello, 2010; Friedman and Kuipers, 2013). The basis for this preference lies in the perception of teachers’ humorous verbal and non-verbal behaviors as informal and friendly. These behaviors challenge the traditional, more serious image of teachers, thereby increasing students’ willingness to communicate with them (Tong and Tsung, 2020). Lu et al. (2023) further argued that the presence of humor in the classroom motivates students to engage in the learning process, particularly because of the perceived high-quality relationship with their teachers. Such friendly interactions serve to diminish the rigid hierarchy typically present between teachers and students, fostering a sense of closer social connection (Daumiller et al., 2019; Savage et al., 2017). For example, Praag et al. (2017) conducted observations and interviews in actual classroom settings and discovered that teachers often utilize humor to critique students and maintain discipline, aiming to preserve the integrity of the teacher-student relationship. Consequently, instructional humor is recognized as an effective strategy for addressing classroom issues within a positive atmosphere (Bell and Pomerantz, 2016). Furthermore, the use of humor in teaching alleviates students’ tension and anxiety, contributing to a constructive and relaxed classroom environment (Berk and Nanda, 1998; Kobayashi and Berge, 2022; Lujan and Dicarlo, 2016). Students report increased willingness to participate in discussions and share their opinions openly, attributing this to a lack of fear of embarrassment in an environment devoid of tension (Abd Ali et al., 2016; Embalzado and Sajampun, 2019). Tsukawaki et al. (2020) found in their study among primary and secondary school students that teachers’ use of affinity humor positively correlates with a harmonious classroom atmosphere. Students perceived the class as enjoyable and well-disciplined, with a sense of mutual respect among peers. Furthermore, humor that is relevant to the lecture content and materials is favored by students (Liu et al., 2017). Such related humor fosters satisfying social relationships and an entertaining class atmosphere, enhancing students’ enjoyment (Bieg et al., 2022; Bieg et al., 2017; Lu et al., 2023). In contrast, unrelated humor, which does not connect with the subject matter, can lead to a negative classroom atmosphere characterized by anxiety and boredom (Bieg et al., 2017; Tsukawaki and Imura, 2019), as students often find this type of humor uninteresting and perceive it as a failed attempt by the teacher to establish rapport (Dobransky and Frymier, 2004). St-Amand et al. (2023) also found that related humor, as opposed to unrelated humor, enhances students’ sense of belonging at school and emotional wellbeing. Additionally, self-disparaging humor can create a welcoming and enjoyable classroom environment (Luo and Zhan, 2021; Bieg et al., 2017) and is positively linked to strong teacher-student relationships (Bieg and Dresel, 2018). However, overuse of self-deprecating humor may undermine the teacher’s credibility and authority in the eyes of students (Gorham and Christophel, 1990; Wanzer et al., 2006).

##### 4.2.2.2 Learning interest and motivation

The positive emotions generated by a tension-free environment significantly enhance students’ motivation for active learning (Abraham et al., 2014). Thus, researchers have explored how instructional humor impacts students’ learning interests and motivation on an individual level. Studies indicate that students engaged in courses where humor is a component show increased motivation and a heightened interest in learning (Davis and Arend, 2013; McKeachie and Svinicki, 2006). Instructional strategies that incorporate humor, such as jokes and entertaining actions, stimulate students’ enthusiasm and participation in educational activities (Vijay, 2014). For instance, Tong and Tsung (2020) conducted interviews in second-language classrooms and reported that the natural integration of humor in lessons boosted students’ motivation to learn and use a foreign language. This beneficial effect of instructional humor on student motivation and interest is also evident in online learning environments, including video-based and gamified learning platforms, where it particularly re-engages initially disinterested audiences (Cook et al., 2023; Wegener, 2022). Moreover, researchers are almost in favor of the positive function of instructional humor that is associated with courses and materials when mentioning different types. Humor related to course content significantly boosts students’ willingness to participate and enhances their learning motivation (Bieg and Dresel, 2018; Luo and Zhan, 2021; Machlev and Karlin, 2017; Tsukawaki and Imura, 2019), fostering affective engagement and motivational behaviors in the classroom (St-Amand et al., 2023). This motivational benefit is notably crucial for students who initially exhibit low interest (Matarazzo et al., 2010). These findings are supported by a longitudinal study, which presents evidence of the long-term benefits of content-relevant humor (Masek et al., 2019). Students exposed to related humor over a 14-week course reported increased interest in learning and a more active motivation to participate. Conversely, the effects of humor unrelated to course content remain debated. Some researchers argue that unrelated humor can stimulate students’ motivation, interests, and enthusiasm (Luo and Zhan, 2021; Pekrun and Linnenbrink-Garcia, 2012; Wanzer et al., 2006), while others contend it does not effectively enhance motivation or engagement, despite its positive emotional impact (Luo et al., 2023; Wang and Chen, 2022). Furthermore, several studies suggest that unrelated humor may actually detract from students’ interest and motivation (Bieg and Dresel, 2018; Gao et al., 2022), particularly leading to boredom and disengagement when overused (Bieg et al., 2017; Machlev and Karlin, 2017; St-Amand et al., 2023). The primary goal of unrelated humor, such as jokes or stories, is only to amuse, leaving students unable to experience these humorous moments firsthand (Bieg et al., 2019) and may lead them to view these attempts at humor as time-wasting and ineffective in alleviating boredom or enhancing engagement.

In summary, there is a consensus among existing studies that the appropriate use of instructional humor enhances teacher-student relationships and the classroom atmosphere, leading to students’ positive attitudes toward class, such as increased motivation and interest. These findings are primarily supported by self-report questionnaires and interviews across numerous empirical studies. However, several areas require further investigation in future research. Firstly, the excessive use of instructional humor may erode the necessary boundaries within teacher-student relationships, pushing the informal instructional approach beyond acceptable limits (Zhang, 2005). It is important to delineate the boundaries for using instructional humor, especially types that do not contribute to the clarification and elaboration of knowledge, such as unrelated humor and self-disparaging humor. Secondly, findings regarding the impact of instructional humor, particularly unrelated humor, on students’ affective performance remain inconsistent. More empirical evidence, especially from experimental studies, is necessary to elucidate the non-cognitive effects of humor in the classroom. Additionally, research has demonstrated a correlation between individuals’ affective responses to humor and the activation of specific brain cortexes (Kohn et al., 2011; Moran et al., 2004). Cognitive neural technologies, such as ERP and fNIRS, could be employed to objectively assess students’ affective performance, addressing the limitations and biases inherent in previous self-report surveys. Finally, students’ perceptions of humor are influenced by individual differences, such as their sense of humor, humor style, and social background, which in turn affects their views on teachers and the class (Lu et al., 2023; Praag et al., 2017; Wu et al., 2023). Future research should focus on understanding how students’ characteristics play a role in the impact of humor on effective learning outcomes.

## 5 The summary of existing research

The concept of “humor” has evolved significantly from its original medical connotation to its current widespread understanding as something funny and entertaining. This shift has broadened the application of humor beyond specific fields, incorporating it into educational contexts. However, the nature and demands of instructional settings impose certain limitations on the use of humor. For instance, humor that involves racist or sexual content, while potentially amusing in a talk show setting, is inappropriate for the classroom. The relationship between humor and instruction dictates the suitable types of humor for educational environments. Teachers might employ humor to enhance learning, such as devising humorous examples to clarify course material, sharing amusing anecdotes, or engaging in self-deprecating humor to connect with students. Furthermore, the application of humor in teaching is influenced by individual differences among teachers, such as personality and experience, leading to varied humor styles in the classroom. Yet, educators must also be mindful of their students’ diverse characteristics to prevent misunderstandings. In essence, instructional humor can be effectively integrated into any subject or course, provided it is applied judiciously and appropriately.

Importantly, the use of instructional humor in educational settings serves specific pedagogical purposes for teachers. The clearest and most consistent empirical finding regarding instructional humor in educational settings is its affective effects on students. Teachers can employ humor to foster a welcoming classroom environment and strengthen their relationships with students. This positive affective experience can, in turn, boost student engagement and participation. Crucially, the benefits of instructional humor depend on its appropriate use; humor that disparages others, for example, can have detrimental effects on students’ affective responses. Moreover, excessive or content-irrelevant humor may undermine students’ positive engagement, leading to boredom. The impact of instructional humor on cognitive learning outcomes presents a more complex picture. There is significant empirical support for the notion that well-integrated humor, especially humor related to course content, can enhance both perceived and actual learning outcomes, which is supported by correlational studies and pedagogical experiments linking humor in learning contexts with improved self-assessed learning outcomes and exam performance. Conversely, some research contends that humor does not significantly affect learning achievements or may even obstruct them, citing a lack of evidence for humor’s role in enhancing knowledge acquisition and recall. Investigations into the cognitive processes involved in learning with humor have also yielded mixed results. While some studies suggest that humor can enhance attention and cognitive elaboration, facilitating learning, others indicate that it may distract from these processes. The conflicting findings on whether humor aids or hinders cognitive engagement highlight the need for further research to clarify its role in educational achievements.

## 6 The direction of future work

Investigating humor usage by teachers in educational settings reveals significant variability in approaches and preferences, including the frequency of humor attempts in classrooms. Previous studies, such as those by Masek et al. (2019) and Ziv (1988), have suggested an optimal humor frequency of three to four instances per class session. However, it is important to note that the ideal frequency may vary depending on the subject matter and the instructional format, indicating a need for future research to further investigate the relationship between humor frequency and various learning contexts, especially considering the rise of digital learning platforms and the unique dynamics they introduce compared to traditional classroom settings. Moreover, the effectiveness of instructional humor is influenced by individual differences among both teachers and students, including gender and cultural background. Some teachers may naturally excel in integrating humor into their teaching, while others may find it challenging. Similarly, students’ perceptions of humor can vary widely, influenced by their personal traits and cultural contexts, which underscores the importance of adapting humor to fit both the educator’s style and the students’ backgrounds, highlighting the necessity for additional research to understand the moderating effects of these individual factors on the outcomes of humor in education. Humor, as a form of communication, can be developed like other social skills (Booth-Butterfield and Wanzer, 2010). Research suggests that the manner in which teachers employ humor is also influenced by their level of teaching experience (e.g., Downs et al., 1988). This implies that the ability to effectively integrate humor into teaching is a professional competency that can be enhanced. Consequently, there is a compelling argument for investigating whether targeted training can improve teachers’ use of instructional humor, optimizing its positive impacts on educational outcomes.

Another area requiring further research is the impact of instructional humor on learning. Current studies predominantly support the notion that humor influences both cognitive and affective aspects of student learning, as evidenced by self-report questionnaires. However, the educational advantages of instructional humor present contradictions that need resolution in future investigations. Primarily, findings regarding the cognitive impact of instructional humor are inconsistent. There is a positive correlation between humor related to course content, as utilized by instructors, and students’ perceived learning effectiveness. Conversely, the influence of other forms of appropriate humor on learning outcomes remains unclear. The complexity increases when considering the actual learning achievements linked to the use of instructional humor. Researchers have not reached an agreement on whether humor, including humor related to course content, positively or negatively affects student performance on tests. According to the IHPT theory proposed by Wanzer et al. (2010), humor can facilitate knowledge acquisition and recall by enhancing student attention and elaboration. However, few studies have focused on these aspects, and the available results are inconsistent. This is largely because these conclusions are based on self-reports collected post-learning, which may not accurately capture students’ cognitive processes during learning and information processing. Thus, there is a critical need for research that objectively and immediately measures students’ cognitive activities when exposed to different types of instructional humor to elucidate its effects on attention and information elaboration. The latest neural evidence could enhance our understanding of the cognitive function of humor and resolve the conflicting arguments regarding its effects on cognitive learning performance. In addition, the consensus in existing research on humor’s affective function is nearly consistent. Scholars largely agree that course-related humor positively predicts a relaxing classroom atmosphere and comfortable teacher-student relationships, further motivating students in their learning. Conversely, humor that is unrelated or self-deprecating is not as effective and may even cause students to feel anxious and bored when overused. Despite this agreement, more empirical evidence is needed to define the necessary limits of humor, especially those unrelated types, to maximize its affective function in learning. Moreover, experimental research conducted in natural classroom settings is less common, with most being cross-sectional. Longitudinal research, similar to the work of Ziv (1988) and Masek et al. (2019), is required to provide evidence for the long-term effects of instructional humor on learning and support the use of humor in practical instruction. Taken together, experimental research conducted both in natural classroom settings and laboratories through controlled interventions of humor attempts is urgently needed to define the causal relationship between humor and learning and to improve upon existing conclusions drawn from questionnaires.

Finally, further evidence on theoretical insights into the application of humor in educational settings and its impact on learning outcomes could contribute significantly to the field of instructional humor. The IHPT concept proposed by Wanzer et al. (2010) is a foundational contribution in this area, with preliminary validation provided by the study conducted by Tsukawaki and Imura (2019). Nevertheless, alternative perspectives have been offered by scholars such as Bieg and Dresel (2018) and Bolkan and Goodboy (2015), reflecting a diversity of opinions and theoretical approaches. This divergence is largely due to differing views on the cognitive and affective roles of instructional humor. Recognizing the intricacies of humor within educational contexts is crucial, particularly given its effectiveness is moderated by a variety of factors. To refine the theoretical framework surrounding instructional humor, it is imperative to incorporate and consider a broader array of variables and theories. Such an endeavor will not only clarify the specific impacts of humor on learning but also enhance pedagogical strategies involving humor, thereby enriching both teaching and learning experiences.

## 7 Conclusion and implication

The present study performed a structured review of existing literature on instructional humor and provided a detailed discussion of their theoretical and practical contribution through compiling related findings. Significantly, we comprehensively summarized and evaluated the overall efficacy of adopting humor in educational settings, highlighting the current challenges faced by educators in fully using humor to enhance educational effectiveness because of the research inconsistences. According to the extant gaps of previous articles, this paper further identified areas for future nuanced research within this field to refine the strategies for incorporating instructional humor into educational practices effectively. Overall speaking, as the five-decade literatures on the topic of instructional humor developing rapidly with a wealth of academic achievements, this review study, helped to advance our integral knowledge of instructional humor and further contribute to teaching and learning efficiency of adopting such method. Based on the summary of relative literatures on instructional humor, despite occasional inconsistencies, the current study recommended advisable guidance for the humor usage in class and offer valuable insights into strategies for enhancing the positive effects of instructional humor.

First, instructional humor serves as a beneficial tool, though it is not a prerequisite for effective classroom interaction. Teachers are encouraged to employ humor in a manner that aligns with their comfort and disposition. For instance, educators with a lower inclination toward humor may choose to minimize their use of humorous attempts during their courses, which is deemed acceptable, especially if attempting spontaneous humor feels forced or burdensome. Nonetheless, these educators can still incorporate humor into their lessons through the use of pre-planned humor, such as showing humorous videos or incorporating amusing images into presentations. Moreover, the application of humor is considered a professional skill that varies with a teacher’s experience. As such, educators are advised to continually refine their ability to integrate humor into their teaching, thereby maximizing the educational benefits of instructional humor.

Second, existing research confirms that humor must be perceived positively by students as a prerequisite for its beneficial effects in instructional contexts. Teachers should avoid using inappropriate humor, especially when it disparages students. The aim of incorporating humor in the classroom is to create laughter with students, not at their expense. Thus, hostile and anti-affiliative humorous behaviors are inappropriate, as they potentially lead to anxiety and a disharmonious classroom atmosphere. Furthermore, teachers must thoroughly consider the characteristics and backgrounds of their students before employing humor to prevent confusion and misunderstandings. For instance, students from different cultures may react differently to the same humorous behavior, including humor itself. Additionally, most related studies focus on university students because older audiences typically have a more developed sense of humor than children and can more easily understand the nuanced meanings behind humor’s outward expressions (Machlev and Karlin, 2017). Therefore, teachers need to select specific types of humor based on their students’ ages, particularly when teaching younger children who might not fully comprehend the humor and could receive inaccurate information. In summary, incorrectly using humor in the classroom can be more detrimental than not using humor at all.

Third and most important, instructional humor offers both affective and cognitive benefits in educational contexts. When used appropriately, humor can enhance students’ positive emotions, fostering warm relationships and a conducive atmosphere, which in turn, enhances their motivation and engagement. However, it is essential to be mindful of the potential negative impacts of excessive humor use in the classroom, which can undermine instructors’ credibility and lead to student disengagement (Bruschke and Gartner, 1991). Echoing Aristotle’s notion that “thinking begins with questions and surprises,” laughter acts as a catalyst for active and focused thinking rather than being the ultimate objective of instructional humor. Thus, educators’ humor efforts should aim to increase students’ motivation and their ability to engage deeply with the material (Wanzer et al., 2010). Most studies support the use of course-related humor, such as humorously illustrating a concept being taught. This approach aids in enhancing students’ memory and recall of learning content, given the mind’s tendency to prioritize the retrieval of humorous information over non-humorous content (Schmidt, 2002). Moreover, the additional attention and cognitive resources devoted to processing humor do not detract from learning; rather, since the humorous content is related to the concepts being taught, it helps reinforce understanding and elaborating. Thus, instructional humor can improve students’ self-assessment of their learning performance and actual knowledge acquisition.
